# A New Doped Graphene-Based Catalyst for Hydrogen Evolution Reaction Under Low-Electrolyte Concentration and Biomass-Rich Environments

**DOI:** 10.1021/acs.energyfuels.4c06084

**Published:** 2025-02-26

**Authors:** I. Vidal-Barreiro, P. Sánchez, A. de Lucas-Consuegra, A. Romero

**Affiliations:** † Department of Chemical Engineering, School of Chemical Sciences and Technologies, University of Castilla-La Mancha, Avda. Camilo José Cela 12, Ciudad Real E-13071, Spain; ‡ Department of Chemical Engineering, Higher Technical School of Agronomical Engineers, University of Castilla-La Mancha, Ronda de Calatrava 7, Ciudad Real E-13071, Spain

## Abstract

Graphene-based catalysts are emerging as promising alternatives to reduce reliance on metal-based catalysts in the hydrogen evolution reaction (HER). This study introduces a novel family of metal-free graphene-based materials codoped with nitrogen and phosphorus (GNP). These materials were synthesized, characterized, and evaluated for HER performance as glucose-tolerant cathodes for biomass electrolysis in a soft alkaline medium, referred to as Mixed Electrolyte (ME): 0.1 M NaOH + 1.0 M Na_2_SO_4_. It was found that the calcination time directly affects the catalytic properties of the final catalysts, with longer calcination times enhancing HER activity. This was attributed to the effective incorporation of nitrogen (N pyrrolic, N quaternary) and phosphorus (P graphitic) into the graphitic network, along with increased catalyst mesoporosity, which significantly improves mass and electron transfer. Furthermore, chronopotentiometry tests revealed substantial electrochemical activation of HER catalytic performance, stemming from the removal of heteroatoms from the carbon framework. This process, confirmed by XPS and Raman Spectroscopy, led to the formation of topological 5- and 7-membered carbon rings, which serve as the main active sites for the reaction. This significantly accelerates the water dissociation activity, leading to improved catalytic performance with a final overpotential (η_10_) of −0.386 V in ME. Notably, the exceptional stability and electrochemical activity under various alkaline media, along with its tolerance in the presence of glucose, make this new cathodic catalyst a suitable candidate for a membrane-less biomass electrolyzer.

## Graphical abstract

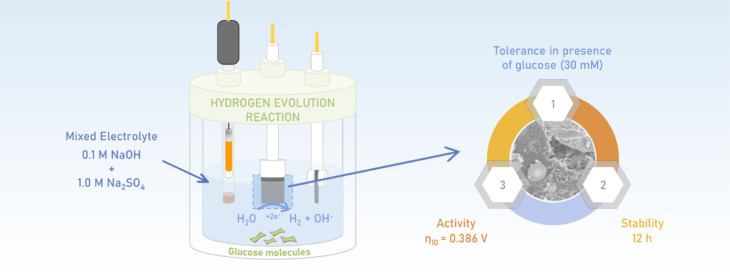

## 1 Introduction

Although the consumption of fossil fuels began in the Industrial Revolution, several decades of scientific and technological advancements, population growth, and higher living standards have contributed to their depletion, projected by 2060, and an increasingly evident climate change.^1,2^ To overcome the critical energy and environmental situation and reverse the trend, new renewable alternatives must be explored^3,4^

Hydrogen,^5,6^ the most abundant element with a high energy density of 140 MJ/kg, is currently in the spotlight. Therefore, the development of advanced technologies is crucial to harness its potential as a promising feedstock and energy carrier. A color classification system^7,8^ has been established according to the type of technology, starting reagents, and environmental impact, with particular emphasis on water electrolysis^9,10^ as an electrochemical technique capable of generating green hydrogen (H_2_) by splitting water (H_2_O) molecules without emissions. The reduction of water to hydrogen, also known as the hydrogen evolution reaction (HER), takes place in the cathode of the electrolyzer in either acidic (2 H^+^_(aq)_ + 2 e^–^ → H_2(g)_) or alkaline (2 H_2_O_(aq)_ + 2e^–^ → H_2(g)_ + 2 OH^–^_(aq)_) medium. However, its efficiency is hindered by inherent thermodynamic and kinetic challenges of the anodic oxygen evolution reaction (OER), resulting in significant overpotentials.^11–13^

Replacing the oxygen evolution reaction (OER) with the favorable oxidation of a biomass molecule, such as glucose, could enhance hydrogen production efficiency and simultaneously generate high-value-added organic molecules, while also reducing the basicity of the reaction media.^14,15^ Indeed, this system could potentially eliminate the need for a membrane, as hydrogen (H_2_) is the only gas produced in the electrolyzer,^16^ which can be easily separated from the liquid electro-oxidation products. This is the objective of the European ELOBIO project (Electrolysis of Biomass), which aims to work with various platform biomass molecules, including glucose and 5-hydroxymethylfurfural (5-HMF). Hence, new cathodic catalysts should be developed that demonstrate high electrocatalytic activity at low electrolyte concentrations (ME: 0.1 M NaOH + 1.0 M Na_2_SO_4_), ensuring the stability of platform biomass molecules and high electrocatalytic stability in the presence of these molecules (glucose).

In this context, an attractive and promising approach involves the use of metal-free carbon-based catalysts,^17^ such as porous carbon,^18^ nanotubes,^19^ nanofibers,^20^ fullerenes,^21^ graphene^22^ and its derivatives.^23^ This last family of carbon allotropes, characterized by sp^2^-hybridized 2D honeycomb lattice of carbon, exhibits high specific surface area, tunable molecular structure, excellent electronic properties, and tolerance to acidic/alkaline media, all of which are essential features to promote the HER reaction.^24,25^ Doping with heteroatoms (such as N, S, P, or B) is recognized as the most effective strategy to introduce multiple active sites. The mechanism involves the creation of a unique electronic structure where the electronegativities of the doped sites differ significantly from those of the carbon structure. The well-known “synergistic effect” ^25–27^ between heteroatoms induces an asymmetric charge redistribution on neighboring carbon atoms, resulting in extremely active sites, often surpassing those of doped graphene.

Theoretical and experimental research has revealed that graphene codoped with nitrogen (N) and phosphorus (P) exhibits significantly enhanced electrocatalytic activity compared to different heteroatom combinations or single-atom doping, achieving performance levels similar to certain traditional metallic catalysts.^26^ This fact is illustrated in the study by Shinde et al.,^28^ where they successfully synthesized metal-free hybrid catalysts based on N and P-doped graphene oxide and graphitic carbon nitride (g-C_3_N_4_), which are highly active for the HER. With an overpotential of −0.340 V at 10 mA·cm^–1^ (η_10_), the remarkable efficiency of these catalysts was attributed to the strong synergistic effect between the exposed active sites in nanoporous graphene and the coupling with g-C_3_N_4_.

In this study, a novel family of graphene-based materials codoped with nitrogen and phosphorus (GNP) was synthesized by heating a mixture of phytic acid (PA), polyethylenimine (PEI), and graphite oxide (GrO) at 900 °C. During this thermal process, N and P heteroatoms were anchored to the carbon matrix while oxygenated groups from GrO were removed, resulting in reduced graphene oxide codoped with N and P. In addition, the influence of calcination time was evaluated in order to improve the HER electrocatalytic performance, with optimal results achieved at extended calcination periods of 10 h. The electrocatalytic experiments also showed an interesting activation during the operation time, which was also investigated in the present work. The results obtained indicate a significant potential for the developed carbon materials to be used as tolerant cathodes in membrane-less biomass electrolyzers.

## 2 Experimental Section

### 2.1 Materials

Graphite powder (99% purity, Ø < 20 μm), potassium permanganate (KMnO_4_), sulfuric acid (96–98%, H_2_SO_4_), phosphoric acid (85%, H_3_PO_4_), hydrochloric acid (≥37%, HCl), phytic acid (50% in H_2_O and M_W_ 600, PA), and polyethylenimine (50% in H_2_O and M_W_ 60 000, PEI) were supplied by Sigma-Aldrich. Ethanol (99.5%, CH_3_–CH_2_OH) and hydrogen peroxide (33%, H_2_O_2_) were supplied by Panreac. Deionized (DI) water was used during all of the synthesis experiments.

### 2.2 Synthesis of Graphene-Based Catalysts Codoped with N and P (GNPs)

#### 2.2.1 Graphite Oxidation

Graphite oxide (GrO) was obtained using an optimized Improved Hummer’s Method.^29^ In a typical synthesis procedure, a mixture of 45 g of KMnO_4_ and 15 g of graphite was slowly added to 400 mL of H_2_SO_4_ under constant stirring for 3 h at 50 °C. To halt the oxidation reactions, 3 mL of H_2_O_2_ and 400 g of flake ice were added to the mixture. Subsequently, the mixture was filtered under vacuum and washed with 200 mL of deionized water, ethanol, and HCl to eliminate the nonoxidized graphite and metal ions and to facilitate the drying process, respectively. Finally, the obtained solid was dried for 3 h at 60 °C.

#### 2.2.2 Synthesis of GNP Catalyst

The preparation of the GNP catalysts involved mixing, vacuum drying, and a calcination step at 900 °C, as shown in Figure 1.

**Figure 1:**
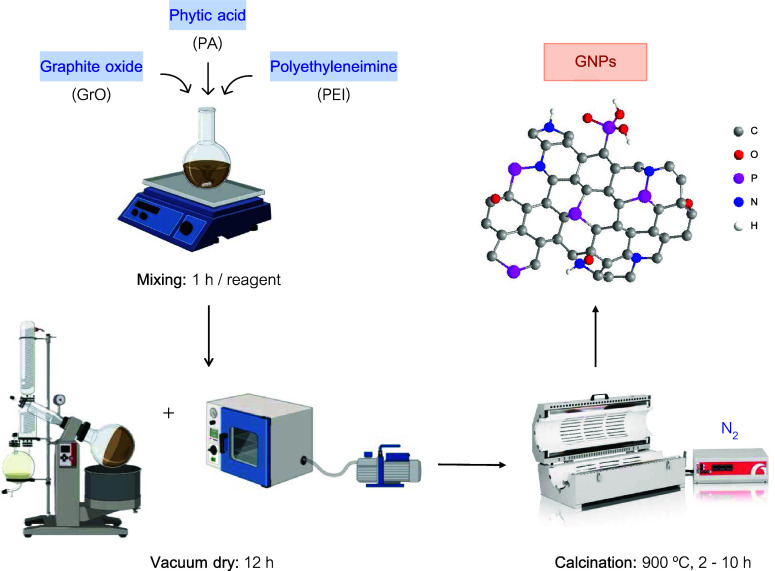
Schematic representation for the synthesis of GNP catalysts.

500 mg of GrO, 6 mL of PA, and 325 mg of PEI were simultaneously added to 300 mL of deionized water (DI), mixing for 1 h per reagent. The obtained precipitate (GNP) was dried in a rotary evaporator (60 °C) and vacuum oven (40 °C), and finally, the sticky material was pyrolyzed at 900 °C for different times at a heating rate of 5 °C·min^–1^ under N_2_ atmosphere, to synthesize the graphene-based catalysts codoped with N and P. In this work, all the products were denoted as GNP–X, where X represents the calcination time. For comparison purposes, catalysts doped with a single heteroatom, either phosphorus (GP) or nitrogen (GN), were also prepared following the same procedure. Note that a maximum calcination time of 10 h was established to ensure the viability and applicability of the catalyst synthesis method, avoiding long synthesis times.

### 2.3 Physicochemical Characterization

N_2_ adsorption–desorption isotherms at 77 K were performed in a QUADRASORB 3SI instrument (Quantachrome), where samples were degassed under vacuum conditions for 6 h at 180 °C before adsorption. The specific surface area was determined using the BET method, while the micropore volume was estimated using the *t*- Plot method.

X-ray Powder Diffraction (XRD) patterns were recorded with a Philips X’Pert MPD diffractometer equipped with cofiltered Cu–Kα radiation (λ = 1.54056 Å) to determine the structural parameters, scanning in the range 2θ = 10–80° (scan rate = 0.02°·step^–1^ using a 4 s·step^–1^). Diffraction patterns were fitted to the Joint Committee on Powder Diffraction Standards (JCPDS).

Crystal morphology and microstructure were analyzed by Scanning Electron Microscopy (SEM) using a ZEISS Gemini SEM 500 FE-SEM with a PIN-diode BSE detector. This instrument was equipped with an energy-dispersive X-ray spectroscopy (EDX) analyzer to confirm the composition of the samples.

The P content in the catalysts was measured by inductively coupled plasma atomic emission spectrometry (ICP-AES). Elemental analyses were performed using LECO CHNS-932 equipment. During the analysis, the sample underwent total oxidation through an instantaneous and complete combustion. Then, the amounts of C, H, and N were measured, and the oxygen groups were calculated by difference.

X-ray photoelectron spectroscopy (XPS) measurements were performed on both catalyst powder and electrodes (fresh and used electrodes prepared by air-spray deposition of powder ink on GDL paper) using a Thermo Scientific Multilab 2000 spectrometer fitted with a 110-mm hemispherical sector analyzer and a dual-anode X-ray source (Al K alpha and Mg K alpha with photon energies 1486.7 and 1253.6 eV, respectively). Spectra were recorded using an analyzer pass energy of 15 eV, an X-ray power of 400 W, and an operating pressure of 10^–9^ mbar. Spectra treatment was performed by using CASA software. Binding energies (BE) were referenced to C *1s* at 284.5 eV. This XPS analysis was performed before and after the electrochemical tests.

Raman spectra were recorded on electrodes by an InVia Renishaw instrument with a 532 nm point-based laser. In all cases, the power density was kept below 1 mW·μm^–2^ to avoid laser heating effects. The resulting spectra (with approximately 30–40 different points on each sample) were fitted with Lorentzian-shaped bands.

### 2.4 Electrocatalytic Activity Measurements

To study the HER activity of the catalysts, a series of electrochemical experiments were carried out in a three-electrode glass cell (i.e., half-cell configuration). This system integrates a glassy carbon rotating disk as the working electrode (Origalys, 5 mm internal diameter), a glassy carbon plate as the counter electrode, and a double junction saturated Hg/HgO (0.1 M KOH) as the reference electrode. Homogeneous catalyst inks were prepared by ultrasonically mixing 2 mg of powder catalyst, 250 μL of water, 750 μL of isopropanol, and 8 μL of Nafion 5 wt %. Subsequently, an aliquot of 19 μL was deposited onto the working electrode to generate a catalytic loading of 0.2 mg·cm^–2^.

Under this configuration, Linear Sweep Voltammetry (LSV) was performed with a rotating speed of 1900 rpm at a 5 mV·s^–1^ scan rate, including a 10-cycle catalyst prestabilization phase, with Tafel slopes obtained from the last LSVs. Stability tests were performed by the chronopotentiometry technique (CP) at different fixed current densities: 10, 20, 30, 50, 20, and 10 mA·cm^–2^ for 2 h per each step. Cyclic voltammetry (CV) was also conducted in order to study the capacitive behavior of the GNP catalysts. To this aim, CV tests were performed at nonfaradaic overpotentials with scan rates of 10, 20, 30, 40, and 50 mV·s^–1^. The plot of current density between the anodic and cathodic sweeps against the scan rate exhibited a linear relationship with its slope equivalent to the double-layer capacitance (Cdl), directly proportional to the material’s electrochemically active surface area.

The GNP-10 h catalyst was also sprayed with an aerograph onto a 1 cm^2^ carbon paper GDL (catalyst loading: 2.5 mg_GNP_·cm^–2^), to understand the electrochemical activation observed during the chronopotentiometry tests. Under this configuration, Linear Sweep Voltammetry and a stability test were also performed.

The electrolytes used in this study were a Mixed Electrolyte (ME: 0.1 M NaOH + 1.0 M Na_2_SO_4_), in the absence or presence of glucose (30 mM), and 1.0 M KOH for comparison purposes with literature. This glucose concentration was selected based on parallel laboratory studies, identifying it as optimal for the anodic oxidation reaction. Given that in a membrane-less electrolysis setup, both the anode and cathode are exposed to the same mixture, the experiments were conducted under these conditions.^30^ All of the electrochemical measurements were taken at room temperature. To keep the solution inert, a continuous flow of ultrapure N_2_ was bubbled for at least 15 min prior to testing the electrodes and then was kept bubbling during the experiments.

## 3 Results and Discussion

### 3.1 Influence of Calcination Time

In the first place, N_2_ adsorption–desorption isotherm measurements were performed to determine the textural properties of the different GNP catalysts. Figure S1 displayed typical IV-type sorption isotherm curves with a rapid uptake at the low relative pressure of *P*/*P*_0_ *<* 0.06, characteristic of microporous material. Moreover, the formation of an adsorption hysteresis at higher pressure (*P*/*P*_0_ = 0.43–0.99) indicates the presence of mesopores in the GNPs, which is beneficial for mass transfer. This was further confirmed by HRSEM images (Figure 2a), where spherical particles of the PA/PEI polymeric complex can be seen interconnected with stacked multiporous sheets. As calcination time increased, so did the sintering of the polymeric particles and the collapse of the porous carbonaceous structure. Although the dramatic reduction in surface area and micropore volume could negatively affect the hydrogen evolution reaction, a greater volume and pore size of mesopores result in an enhancement of the number and accessibility of exposed active sites (Table 1 and Figure 2b).^13^

**Figure 2:**
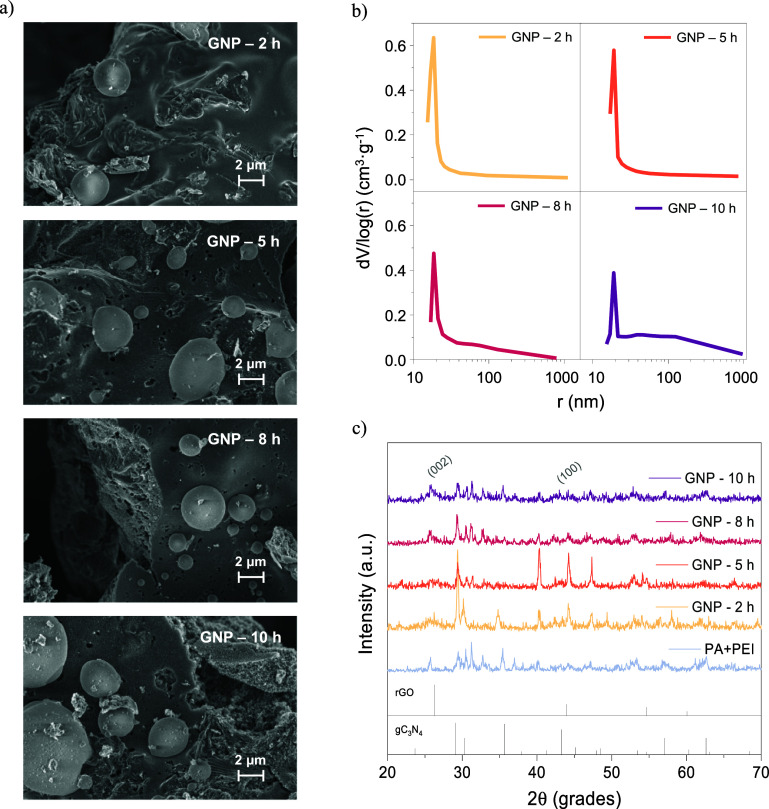
HRSEM images (a), pore size distribution (b), and XRD patterns (c) of GNP catalysts.

**Table 1:** Textural Properties of GNP Catalysts

	BET				
Catalyt	Surface area (m^2^ g^–1^)	Micropore area (m^2^ g^–1^)	BJH mesopore volume (×10^3^cm^3^g^–1^)	T-plot micropore volume (×10^3^ cm^3^ g^–1^)	Average pore size (nm)
GNP-2 h	234	46	112	24	16
GNP-5 h	155	34	93	17	19
GNP-8 h	105	9	106	3	25
GNP-10 h	89	0	152	0	37

Crystallography nature was carefully characterized by X-ray diffraction (XRD). Figure 2c displays the XRD patterns obtained for all of the synthesized materials at different calcination times. All electrocatalysts displayed diffraction peaks around 26° (002) and 43.7° (100), corresponding to the graphite plane and the hexagonal structure of the graphite plane, respectively, both observed in reduced graphene oxide (JCPDS No. 00-003-0401).^31^ The increase in their intensity was typically associated with the graphitization of materials over prolonged calcination times^32^ The remaining signals were attributed to the gC_3_N_4_ (JCPDS Nos. 01-078-1747 and 00-053-0671) and PA/PEI polymerization structures, respectively.^33^

Meanwhile, the chemical composition was investigated through X-ray Photoelectron Spectroscopy (XPS) measurements. Both P and N were identified in each sample, confirming successful doping of the initial graphite oxide (Table 2). Their percentages decreased with prolonged calcination times owing to thermal degradation.^25^ Additionally, as the calcination time increased, the catalyst exhibited progressively higher levels of graphitization. This was evidenced by a rise in the carbon content and a corresponding decrease in the oxygen content. Consequently, the removal of surface hydroxyl groups can generate a higher number of active sites and oxygen vacancies, as shown in Figure S2b, which could also improve HER activity.^34^

**Table 2:** Chemical Composition and XPS Results of GNP Catalysts

	Element content (wt %)				N 1*s* (%)				P 2*p* (%)			
Catalyst	C	O	N	P	N_Pyr_	N_Pyrr_	N_Gr_	N_Ox_	P_0_	P_I_	P_II_	P_III_
GNP-2h	59	33	1	7	20	41	25	14	0	28	57	15
GNP-5h	68	25	1	6	26	37	25	12	3	22	54	21
GNP- 8 h	70	27	1	5	25	29	31	16	5	33	40	22
GNP-10 h	71	24	1	4	20	36	28	16	5	21	40	34

To investigate the bond configuration of each compound, in-depth, high-resolution XPS was also carried out. According to C *1s* spectra shown in Figure S2a, the fitting peaks at 284.5, 285.7, 286.8, 288.4, and 290.1 eV were associated with C–C, C–P/C–O/C–N, C=O, O–C=O, and π–π.^35,36^ Catalyst graphitization resulted in a significant increase of the C–C peak as time increased from 2 to 10 h. Indeed, the presence of the C–P bond proved the successful incorporation of phosphorus into the graphitic network. Three different oxygen species were distinguished in the 1*s* spectra (Figure S2b): structural lattice oxygen (O_I_), OH^–^/defects (O_II_), and electrophilic (O_2_^–^ and/or O^–^) species at binding energies of ca. 528.6, 530.5, and 532.5 eV.^37^ In spite of 10 h of calcination, the signal corresponding to electrophilic (O_2_^–^ and/or O^–^) species became more intense, providing H_2_O anchor sites in the HER.^19^ The N 1*s* spectra (Figure 3a and Table 2) was divided into four peaks at 398.3, 400.1, 401.3, and 402.6 eV related to pyridinic (N_Pyr_), pyrrolic (N_Pyrr_), graphitic (N_Gr_), and oxidized nitrogen (N_Ox_),^38^ respectively. A constant distribution of nitrogen species was observed with a maximum amount of graphitic N at 8 h. The increase in total graphitic N content from 25 at. % in GNP-2 h to 31 at. % and 28 at. % in GNP-8 h, and GNP-10 h, respectively, can only be explained by the conversion of pyridinic and pyrrolic N species into graphitic nitrogen groups at higher calcination times.^39^ Its presence, along with a higher proportion of pyrrolic N, could improve the electron concentration and transport in the carbonaceous structure, thereby enhancing the electrocatalytic activity of the materials.^40^ The slight shift in the P 2*p* spectra peak (Figure 3b) with increasing calcination time may be attributed to changes in the chemical environment, specifically the removal of surface OH^–^ groups.^34^ Nevertheless, this variation remains within the binding energy range of the signal. Four peaks were identified at 131.4, 133.8, 135.2, and 137.0 eV corresponding to graphitic phosphorus (P_0_), oxidized graphitic phosphorus (P_I_), phosphate/pyrophosphate (P_II_), and metaphosphate (P_III_).^41,42^ In this case, the introduction of phosphorus into the graphitic network was related to the appearance of P_0_ peak (reduced species) from 5 h onward, in accordance with the previously mentioned C–P bond.

**Figure 3:**
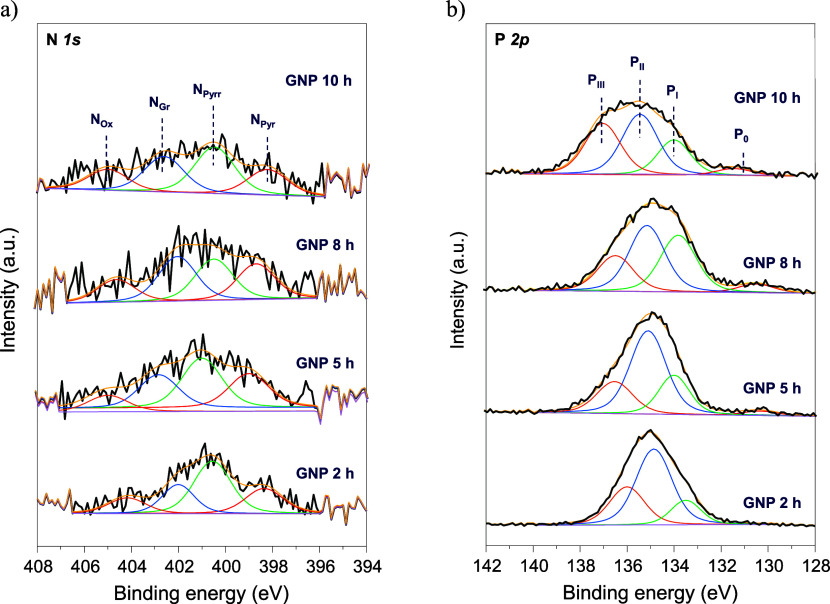
Core level high-resolution N 1*s* (**a**) and P 2*p* (**b**) XPS spectra of the GNP catalysts.

The electrocatalytic activity of GNP catalysts toward the hydrogen evolution reaction (HER) was evaluated using a rotating disk electrode (RDE) in a Mixed Electrolyte solution (ME: 0.1 M NaOH + 1.0 M Na_2_SO_4_), compared with single-doped and commercial Ni/C as reference catalysts. The linear sweep voltammetries shown in Figure 4a demonstrate a direct relationship between calcination time and the electrocatalytic activity of the catalysts, with GNP-10 h exhibiting an overpotential of −0.556 V at 10 mA·cm^–1^ (η_10_). Although it did not achieve the optimal performance of Ni/C in HER, the significant enhancement in electrochemical activity was critically dependent on the presence of both dopants, in comparison with single-doped catalysts. This phenomenon, well-known as the *synergistic effect*,^25–27^ arises from codoping with two different electronegative heteroatoms (N: χ = 3.04, P: χ = 2.19) creating a unique electronic structure with potential catalytically active sites for HER. In this context, the enhanced mesoporosity of the catalyst resulting from extended calcination times improves access for water molecules to active sites, enabling their efficient decomposition into H_2_.^13^ The calculated Tafel slopes (Figure 4b) also confirmed the influence of the calcination time on electrocatalytic activity, being lower at extended periods. An efficient electrocatalyst should present the smallest Tafel slope, indicative of faster reaction kinetics,^43^ which corresponds to the lowest overpotential shown by the GNP-10 h. This can be attributed to faster diffusion of electrolyte ions and easier electronic transport within the unique mesoporous structure of the catalyst.^44^ Additionally, the rate-determining step was identified as the Volmer step (>118.2 mV·dec^–1^) due to the significantly reduced concentration of H^+^ ions in alkaline media, which hinders the dissociation of water molecules.^45^ However, the presence of nitrogen (N_Pyrr_, N_Gr_) and phosphorus (P_0_) in the graphitic network, which act as negative electron centers, facilitates the attraction of H_2_O molecules, thereby promoting the Volmer reaction.^46^

**Figure 4:**
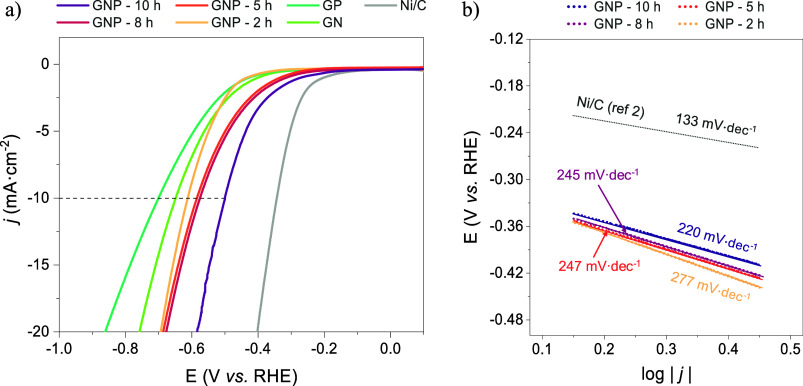
Linear sweep voltammetry (LSV) curves of GNP catalysts (a) and their corresponding Tafel slopes (b) in a Mixed Electrolyte.

The HER stability is another critical parameter for practical applications, assessed using a multistep chronopotentiometry (CP) technique at different current densities (Figure 5a). An initial slight reduction in potential was observed (±30 min), which quickly stabilized and remained constant. In spite of this, each catalyst experienced an activity improvement of 4% (GNP-2 h), 11% (GNP-5 h), 21% (GNP-8 h), and 21% (GNP-10 h) between the initial and final 10 mA·cm^–2^ steps. Their electrochemical active surface area (ECSA), estimated from the double-layer capacitance method (Figures S3 and S4) because of the proportional relationship, revealed that the Cdl value of the catalysts remained unchanged after activation, despite increasing with calcination time as expected (Figure 5b). Therefore, the improvement in electrochemical activity cannot be attributed to a change in the ECSA of the GNPs. The electrocatalytic results demonstrated not only the direct influence of calcination time but also the superior activity of GNP-10 h over the materials before and after the CP test, achieving a final η_10_ of −0.440 V in ME, under a low electrolyte concentration.

**Figure 5:**
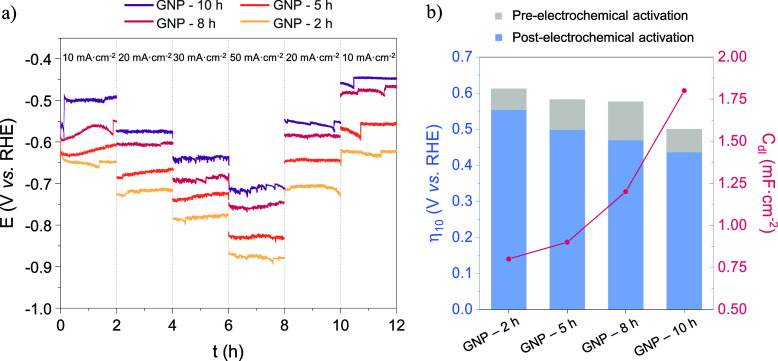
HER stability tests (a) and electrochemical activity (b) of GNP catalysts in a Mixed Electrolyte.

### 3.2 Influence of the Alkaline Media

The stability of the most effective catalyst was also evaluated in different alkaline environments: in the presence of an organic molecule (ME + glucose, 30 mM) to simulate practical applications in biomass electrolysis, and in 1.0 M KOH for comparison with existing literature. It should be noted that GNP-10 h continues to exhibit electrochemical activation after the 12 h CP test, independently of the chosen electrolyte. As shown in Figure 6a,b, the catalyst activity remained consistent regardless of the presence of glucose, indicating promising applicability for a membrane-less biomass electrolyzer. However, as expected, the best results were achieved under strongly alkaline conditions (η_10_ = −0.320 V) due to the high concentration of OH^–^ ions, which enhance the charge transfer from the electrode to the adsorbed reactants, primarily water molecules.^17^

**Figure 6:**
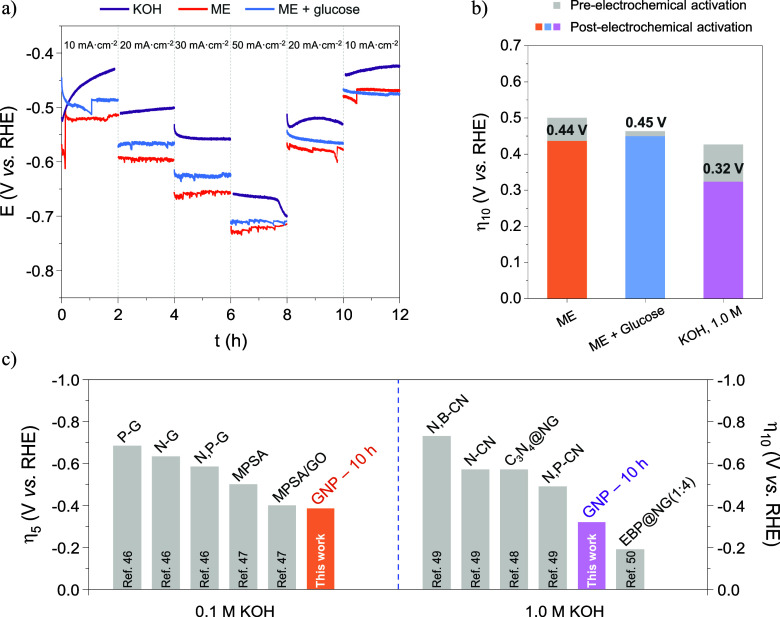
Influence of different alkaline media: KOH 1.0 M and Mixed Electrolyte (without or with glucose) in chronopotentiometry activation (a) and electrochemical activity (b) of GNP-10 h, and comparison of HER activity with carbon-based catalysts available in the literature (c).

The HER activity of the most active catalyst was compared with various carbon-based materials from the literature under similar conditions, as summarized in Figure 6c. The synthesized material, GNP-10 h, achieved the lowest overpotential in mild alkaline media (η_5_ = −0.386 V)^47,48^ under our working conditions. However, this trend does not persist in 1.0 M KOH (η_10_ = −0.320 V).^49,50^ It remains uncertain whether the EBP@NG(1:4) catalyst^51^ would exhibit higher electrochemical activity under OH^–^ low concentration conditions, where charge transfer is limited. Therefore, the GNP-10 h is still regarded as a promising new carbon-based electrocatalyst for both conventional and biomass-assisted electrolysis.

### 3.3 Investigation of Electrochemical Activation during HER

In order to elucidate the behavior of the catalysts during chronopotentiometry at different current densities, the GNP-10 h was air-brush deposited on a 1 cm^2^ carbon paper (GDL), and the experiments were repeated in Mixed Electrolyte (Figure 7a). This method enables the identification of physicochemical changes in the optimal catalyst by comparing the depositions before and after electrochemical activation. Initially, the LSV curves presented in Figure 7c confirmed the reproducibility of the experiment by displaying slightly increased HER activities after the CP test (Figure 7b), in comparison with GNP-10 h (η_10_ = −0.440 V) and comparison of LSVs curves pre- and postactivation (c) in Mixed Electrolyte.

**Figure 7:**
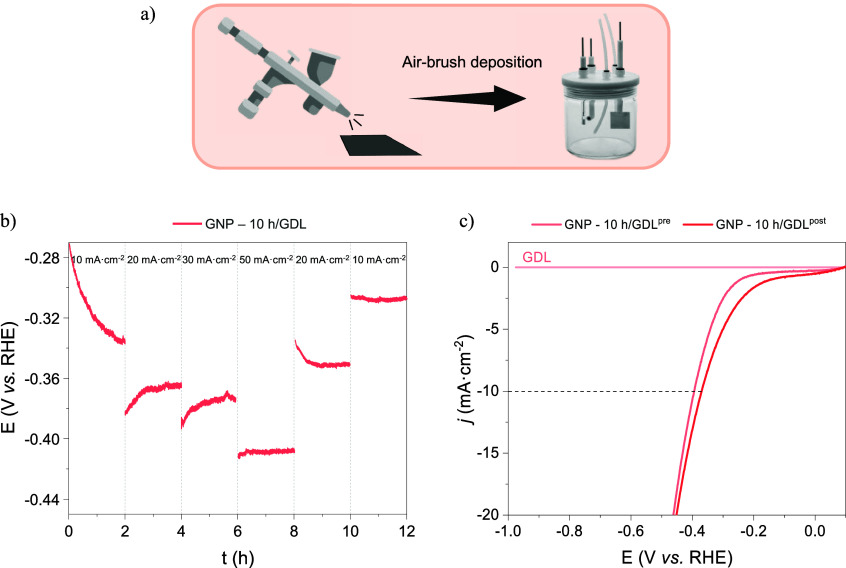
Schematic illustration of GNP-10 h air-brush deposition on carbon GDL (a), HER stability test (b),

No significant differences were observed in the HRSEM images of the initial and activated GNP-10 h (Figure S5), implying that activation does not substantially modify the catalyst morphology. Nevertheless, X-ray photoelectron spectroscopy (XPS) measurements exhibited interesting variations in nitrogen and phosphorus content after electrochemical activation, with levels decreasing from 1 wt % N and 4 wt % P to 0 wt % for both elements (Figure 8a1,a2). In a prior study, Lu et al.^52^ examined the activity enhancement of heteroatom-doped carbons during the hydrogen evolution reaction. This enhancement was attributed to the hydrogenation and subsequent removal of the dopants from the carbon framework after 120 h at a current density of 10 mA·cm^–2^. This process led to the reorganization of carbon atoms into different types of membered rings, creating new defects that serve as the main active sites for HER. Notably, this behavior had been previously reported by Jia et al.,^53^ who suggested that such carbon structural reorganization could minimize the energy of vacancy sites resulting from dopant removal. In both cases, DFT studies not only supported the characterization results obtained via XPS and Raman but also identified the 5- and 7-membered rings as the energetically favorable configurations for enabling the HER.

Hence, the potential structural changes experienced by the catalyst were evaluated by using Raman Spectroscopy. Two main peaks can be distinguished in Figure 8b: the D band (∼1350 cm^–1^), associated with edge dislocations or defects, and the G band (∼1590 cm^–1^), related to the vibrations of sp^2^-bonded carbon atoms.^54,55^ The removal of the doping atoms results in a further increase in defects, as shown by the rise in the *I*_D_/*I*_G_ ratio from 1.11 to 1.14, corroborating the previously stated observations.^52,53^ The new catalyst structure proposed in Figure 8c may facilitate the dissociation of water molecules, thereby enhancing catalytic activity and achieving a final overpotential value (η_10_) of −0.386 V in Mixed Electrolyte.

**Figure 8:**
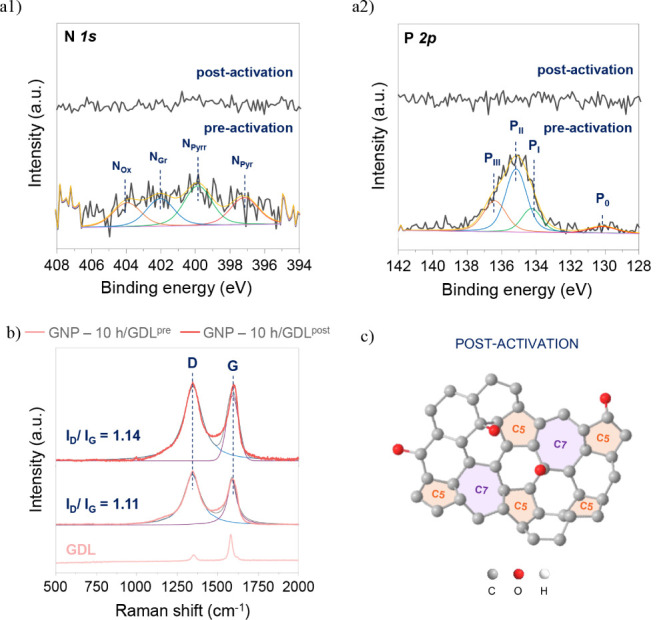
N 1*s* and P 2*p* XPS (a1,a2) and Raman spectra (b) of GNP-10 h/GLD in Mixed Electrolyte, pre- and postelectrochemical activation, and the atomic reorganization proposed based on the literature (c).

## 4 Conclusions

This work has allowed the development of a novel family of graphene-based materials codoped with nitrogen and phosphorus (GNP) as efficient and promising metal-free catalysts for the hydrogen evolution reaction (HER) in biomass electrolysis under soft alkaline conditions. Based on a straightforward and scalable method of synthesis, the calcination time was found to significantly affect the catalytic properties of the GNP catalysts. Prolonged calcination (10 h) provided optimal HER performance, achieving an overpotential of −0.556 V at 10 mA·cm–^2^. It was attributed to the synergistic effect of both N (N pyrrolic, N quaternary) and P (P graphitic), successfully incorporated into the graphitic network, along with the increase in intrinsic mesoporosity and the ECSA of the catalyst.

Furthermore, the electrocatalytic activation observed in each catalyst during chronopotentiometry tests was attributed to the surface removal of heteroatoms from the carbon framework. This phenomenon, confirmed by XPS and Raman Spectroscopy, led to the formation of topological 5- and 7-membered rings, which serve as the main active sites for HER. Both structural transformations and the unique mesoporosity of the materials significantly accelerate water dissociation through efficient electron and mass transfers, thereby enhancing catalytic activity with a final overpotential of −0.386 V. Additionally, with its remarkable stability and activity in various alkaline media (η_10_ = −0.320 V in KOH 1.0M, – 0.440 V in ME), as well as tolerance in the presence of glucose (η_10_ = −0.450 V), the GNP-10 h is positioned as a promising new carbon-based electrocatalyst for both conventional and biomass electrolysis in final applications.

## Supplementary Material



## Data Availability

The data that support the findings of this study are available in Zenodo: 10.5281/zenodo.14354551.

## Notes

N_2_ adsorption–desorption isotherms (Figure S1), C1*s* and O2*s* XPS spectra (Figure S2), cyclic voltammetry curves at different scan rates (Figure S3), linear fitting of Cdl currents vs scan rates of GNP catalysts (Figure S4), HRSEM images of GNP-10 h/GLD pre- and postelectrochemical activation (Figure S5) (PDF)
