# Supplementary material for: A New Doped Graphene-Based Catalyst for Hydrogen Evolution Reaction Under Low-Electrolyte Concentration and Biomass-Rich Environments
Source: Energy Fuels. 2025 Feb 26;39(9):4515–24. doi: 10.1021/acs.energyfuels.4c06084 (PMC12124224; doi:10.1021/acs.energyfuels.4c06084)
Supplement: Supplementary file 1 [file ef4c06084_si_001.pdf]

Supporting Information

A new doped graphene-based catalyst for hydrogen evolution reaction under low-electrolyte concentration and biomass-rich environments

I. Vidal-Barreiro [1*], P. Sánchez [1*], A. de Lucas-Consuegra [1*], A. Romero [2*]

[1] Department of Chemical Engineering, School of Chemical Sciences and Technologies, University of Castilla-La Mancha, Avda. Camilo José Cela 12, E-13071, Ciudad Real, Spain.

[2] Department of Chemical Engineering, Higher Technical School of Agronomical Engineers, University of Castilla-La Mancha, Ronda de Calatrava 7, E-13071, Ciudad Real, Spain.

Corresponding authors: * Antonio.Lconsuegra@uclm.es; amaya.romero@uclm.es

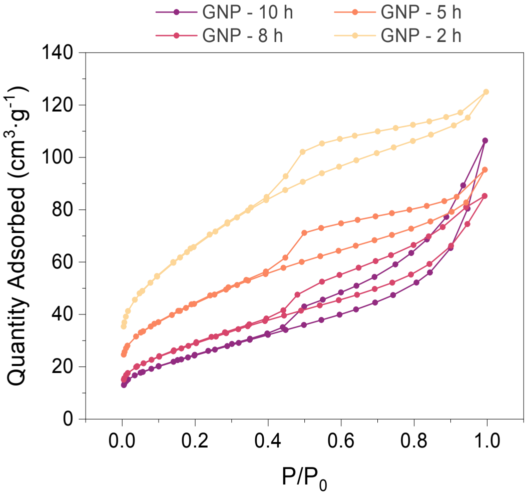

Figure S1. N_2 adsorption–desorption isotherms of GNP catalysts

Table S1. C *1s* and O *1s* XPS results of GNP catalysts

Catalyst	C *1s* (%)					O *1s* (%)		
	C-C	C-P	C=O	O-C=O	π- π	O_I	O_{II}	O_{III}
**GNP – 2 h**	49.0	33.5	11.0	4.2	2.3	33.1	56.3	10.7
**GNP – 5 h**	52.4	4.7	15.0	5.2	2.7	33.4	49.9	16.73
**GNP – 8 h**	63.7	17.3	10.6	5.0	3.4	36.6	45.9	17.5
**GNP – 10 h**	63.3	16.0	12.5	5.2	3	30.1	45.0	24.9

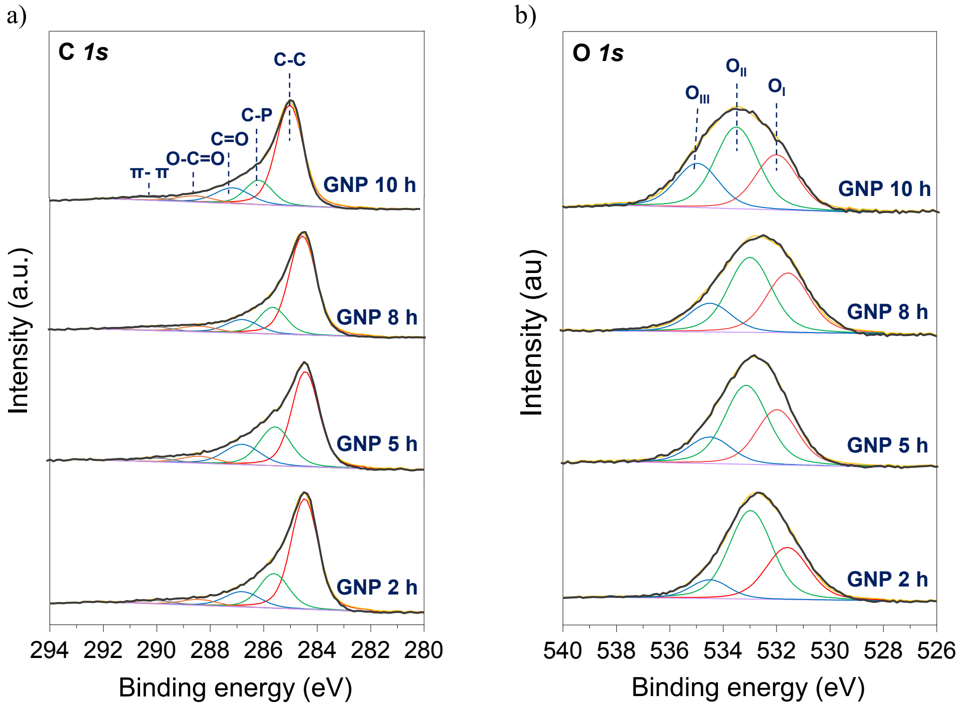

Figure S2. Core level high-resolution C*1s* and O*2s* XPS spectra of GNP catalysts

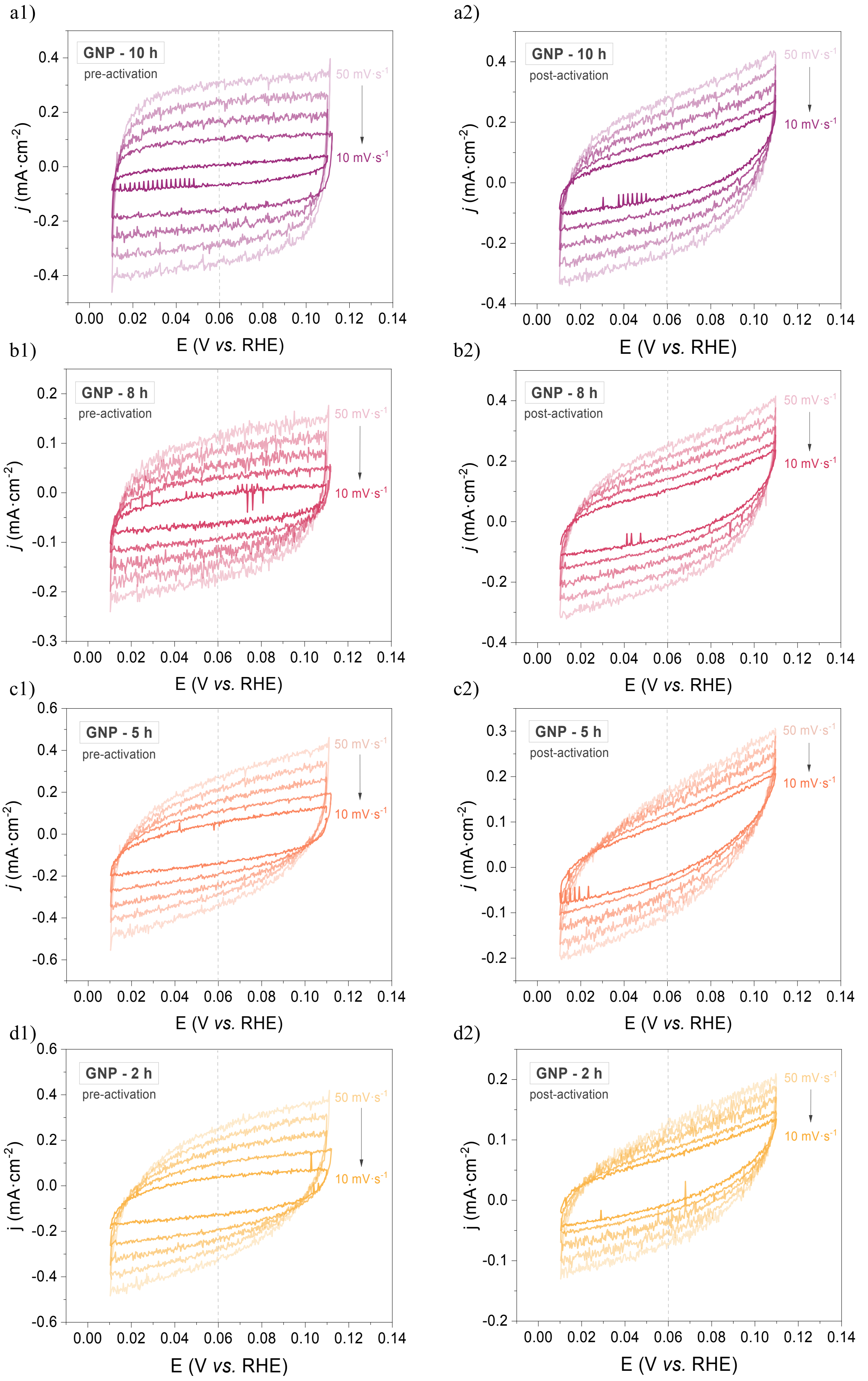

Figure S3. Cyclic voltametry curves at different scan rates (10 to 50 mV·s^{-1}) of GNP catalysts in Mixed Electrolyte,before and after electrochemical activation

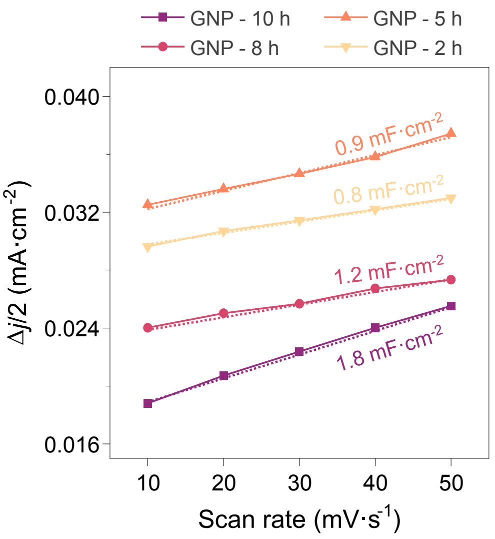

Figure S4. Linear fitting of Cdl currents vs. scan rates of GNP catalysts

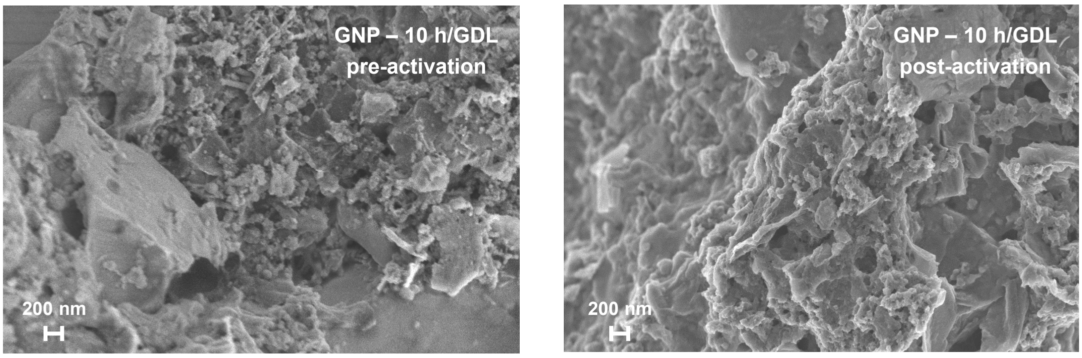

Figure S5. HRSEM images of GNP – 10 h/GLD pre- and post-electrochemical activation
